# The Practitioner's Outlook and Retrospect

**Published:** 1889-10-05

**Authors:** 


### THE PRACTITIONER'S OUTLOOK AND RETROSPECT.

### MEDICINE.

### Gurjon Oil and Leprosy.

Dr. George Foy, surgeon to the Whitworth Hospital, has published a paper* on "The Therapeutic Value of Gurjon Oil on Leprosy." Gurjon oil is yielded by trees of the genus *dipterocarpus*, which are described as majestic trees, attaining a height of from 200 to 300 feet. They grow on the southern slopes of the Himalayas and on the east coast of the Bay of Bengal. It is a dark brown liquid, with a close resemblance in consistence, smell, and taste to balsam of copaiva. The author states that its use was first advocated for leprosy by Surgeon-Major Dougall, medical officer at the Andamans. And he mentions that Dr. Espinet, the medical superintendent of the leper asylum, Trinidad; Dr. Hillis, of the leper asylum, British Guinea; Dr. Khory, of Bombay, and several others have reported favourably. Now, as it happens, Surgeon-Major Dougall did not first advocate the gurjon oil treatment for leprosy, which was proposed by Assistant-Surgeon Arjoon, of Bombay, who, however, kept his treatment a secret for some time. And the authors referred to speak very guardedly about the efficacy of the oil. Espinet says four patients improved as regards their leprous symptoms and general health. Hillis says, "we appear to have a remedy capable of retarding the ravages of the disease, and in some cases apparently curing it." Khory recommends it to promote the healing of leprous ulcers. But the fact is that gurjon oil does *not* cure leprosy. It has been tried extensively in India, and although lepers often improve under its internal and external use, especially when assisted by generous diet, they remain lepers still. It is also questionable if this particular oil is any more efficacious than cod-liver oil. Leprosy is a malady of very slow progress, with deceitful intervals of quiescence, which often occur irrespective of medical treatment, and this amelioration has been often erroneously attributed to gurjon oil and other remedies.

### Causes of Leprosy.

Dr. J. L. Donnet, Inspector-General of Hospitals and Fleets, publishes "Clinical Notes on Leprosy,"† as observed in Lisbon at the Hospital of San Lazero. The two types of leprosy observed are *lepra anæsthetica* and *lepra tuberculosa*. Cases of both forms of leprosy are described, sufficiently typical in their characteristics. With regard to the causes of leprosy, Dr. Donnet is disposed to attribute the disease to peculiar food, for he writes that the majority of the subjects whose cases are related had lived on the sea-board of Portugal, or at no great distance from it, using fish as their chief diet, seldom indulging in the use of flesh meat. Now, such an hypothesis as fish diet causing leprosy cannot be accepted, Mr. Jonathan Hutchinson notwithstanding. It is no more correct than the theory once promulgated, that eating new rice causes cholera. For leprosy prevails in countries far removed from the sea coasts where the people rarely if ever see fish. As regards heredity, Dr. Donnet says he "fails to accept it as the *vera causa* of the disease." But so many instances occur of leprous generations, that heredity must be accepted as a cause of leprosy. In three of the cases detailed by Dr. Donnet there is a family history of leprosy, although Dr. Donnet does not appear to have instituted enquiries further than the father and mother of the sufferers. We have an instance in syphilis of a disease which may be both hereditary and acquired, and this is indeed the case as respects leprosy. Dr. Donnet seems disposed to admit the *bacillus lepræ* as the cause of the malady. Now, whether the bacillus, or any other germ or poison, is the true cause, it is easy to conceive that the germ may be transmitted from parent to offspring. It is also easy to conceive that it may be dormant during a generation. For the excessive minuteness of germs is something inconceivable, no more to be appreciated than the indefiniteness of space. The taint of "black blood," or other peculiarities, may reappear in descendants from even remote ancestors. Acquired leprosy, however, necessitates the transmission of leprous discharge to the abraded skin of a healthy person. This may happen either directly or indirectly in many different ways, when healthy people are in communication with lepers. On the other hand, a healthy person may live for an indefinite period in communication with lepers and not be subjected to the transmission of leprous discharge to an abrasion of his skin. The communication of the disease in this manner is accidental and happens very frequently in tropical countries where the inhabitants are accustomed to walk, at least in the house, without even slippers, and where their feet are more liable to scratches and sores, which may come into contact with leprous discharge containing the germ poison or bacillus of lepra. We have several facts tending to prove that leprosy is communicable by inoculation, the most recent being the inoculation of a criminal at Honolulu with the express purpose of re-demonstrating the fact. Dr. Donnet, in the paper under notice, mentions that the tubercular form of leprosy is known to the Portuguese under the name of *morphœa*. Morphœa, however, is a quite distinct malady, although Dr. Donnet does not tell us this. Morphœa is a special affection of the skin, and has nothing in common with leprosy. It is a fibroid degeneration of the skin. It is indeed, supposed to be a modified form of scleroderma. Discoloured or dingy spots are the characteristic, and from these spots appearing somewhat similar to the spots of *lepra maculosa*, the two maladies have been confused, and leprous spots have been called *morphœa nigra*, or *morphœa rubra*, or *morphœa alba*, according to the colour presenting. But true morphœa is never followed by the general leprous condition.

* *Medical Press*, June 1889. † *British Medical Journal*, August 10, 1889

### Obesity.

A recent medical journal contains a note on a memoir by Dr. Bollinger, of Munich, describing the case of a man who, for the last five years, had suffered from partial obesity. The cheeks, chin, throat, and upper arms exhibited morbid accumulations of adipose tissue. Dr. Bollinger appears to consider this somewhat extraordinary, and, so far as the parts affected are concerned, it may be uncommon. But local obesity of other parts is not unusual. Local accumulation of fat, both internal and external, of the abdomen is frequently met with; and both fatty liver and fatty heart are common complaints. There really seems no valid reason why local obesity should not prevail in any part of the body, although, as a general rule, we are unable to say why this undesirable preference is given to particular parts.

### Aortic Aneurism.

Dr. Gairdner, of Glasgow, details a case* of aneurism of the arch of the aorta perforating into the *vena cava superior*. The symptoms were marked cyanosis with general anasarcous swelling of the upper part of the body. These symptoms were attributed to cold, but on close questioning, the patient admitted having been conscious for two years past of a slight degree of pulsation in the right sterno-clavicular region. The first occurrence of swelling was accompanied by a feeling as if something had given way. There was a loud murmur all over the front of the chest. It is probable the aneurism had existed long before the latter symptoms presented. After the *post-mortem*

* *Lancet*, June 23, 1889.

examination, Dr. Gairdner mentioned that the case was almost unique, although he had seen aneurisms opening into the auricles, pulmonary artery, etc. There are, however, several cases of the kind on record.

### SURGERY.

### Operations for Varix.

In his third lecture on "Varicose Veins of the Lower Extremities,"* Mr. W. H. Bennett, surgeon to St. George's Hospital, deals with certain points of interest, to some of which he has been unable to find reference elsewhere. The treatment of varicose veins by open operation is a method which, although practised centuries ago, has received little encouragement on account of the risks formerly inseparable from it, one of which is that of "blood poisoning." But a better appreciation of the antiseptic system and improved methods of operating have reduced risk to a minimum. As a general rule, the question of the radical treatment does not present itself in varicosity limited to the leg, for here other means are generally sufficient. But when varix involves both the leg and thigh the matter is different. The operations for varicose veins may be divided into three kinds. 1. Those which aim at mere obliteration of the affected vein at one or more points. 2. Those which aim at the removal of portions of dilated vessels. 3. Those which aim at effecting a cure by removal of the whole disease. The strictest precautions as to surgical cleanliness must be observed in all cases, and the part should be packed in wet carbolic dressing for at least four hours before the operation. In the application of a ligature the vein is simply exposed through a small incision, and isolated by gently insinuating an aneurism needle beneath it, by which a ligature is carried round the vessel. In the second operation the vein is exposed for an inch and a half, and two ligatures are applied. The intervening portion of the vessel is then divided. In the removal by dissection of large areas of exaggerated varix, or of isolated varicose masses, a single or crucial incision is made over the mass, and the cellular tissue laid open. Then the whole varix may be quickly laid bare by running a di-vector over and around the dilated vessels. An occasional touch with a scalpel is necessary. As many as possible of the veins running into the varix are now divided, one by one, between double ligatures. The mass is then drawn out and removed. The complete exposure of the varix is much facilitated by throwing the veins into full relief at the commencement, and this is accomplished by a ligature round the limb above the site of lesion. Catgut sutures are usually required to close the wound, and antiseptic dressing is then applied, care being taken to make equable and rather firm pressure. The wound requires little attention after the first dressing, which need not generally be changed for a week, by which time the healing will probably be complete. In his cases, Dr. Bennett has seen neither fever, suppuration, nor other untoward symptoms.

### Hernia.

Mr. C. B. Lockwood, surgeon to the Great Northern Hospital, has recently lectured at the Royal College of Surgeons "On the Morbid Anatomy, Pathology, and Treatment of Hernia." Although so much has been written about hernia, and the subject has been investigated by so many competent surgeons and anatomists, there is still a question at issue, for one school of pathologists believe that in hernia the fault is in the abdominal walls, and others assert that the fault is in the attaching membranes of the intestines. To ascertain if the latter is correct, three main questions are required to be dealt with—viz., the length of the mesentery, the attachment of the mesentery, and the range of movement the mesentery permits to the intestines. It appears that all these conditions are very variable. On such and other grounds the lecturer concludes that in ordinary cases of congenital hernia at least the fault is in the abdominal walls. It would also appear that most cases of acquired hernia are also due to lack of resistance of the abdominal walls. But when there is a large excursion of the intestines, or, in other words, a large hernia, it is associated with a low attachment of the mesentery.

* *British Medical Journal*, Aug. 10, 1889.

### Traumatic Subclavian Aneurisn

Dr. J. Lewtas, of the Indian Medical Service, reports a case presenting at the Murdan Hospital, Punjaub, of "traumatic subclavian aneurism," treated by ligature of both the innominate and carotid arteries.* Consequent on the bursting of a gun, a piece of the breech was supposed to have lodged in the neck just above the right clavicle. The out] line of the clavicle was obscured by a large swelling, hard to the touch, and without pulsation. At first Dr. Lewtas was under the impression that he had to deal with an abscess, and introducing a probe into the original wound, which had not yet healed, he failed to detect any foreign substance. The wound was then enlarged sufficiently to admit the index finger, and a hard substance was felt, which on removal with a pair of forceps proved to be an irregular fragment of metal weighing three drachms. This was followed by an alarming rush of blood. On passing the finger into the wound, Dr. Lewtas found an opening in the subclavian artery behind the *scalenus anticus* muscle. But the man's condition was now desperate from loss of blood, and any attempt to tie the artery at the seat of injury appeared clearly inadmissible. There was not much time for deliberation, and Dr. Lewtas immediately decided to tie the common carotid and innominate arteries. Confiding the control of the bleeding orifice to an assistant, Dr. Lewtas made an incision along the inner border of the lower end of the right sterno-mastoid muscle, notched the outer borders of the sterno-hyoid and sterno-thyroid, and passed a catgut ligature round the common carotid, leaving the ends of the ligature long, so that by drawing upon them he might be guided to the position of the innominate. Using a couple of blunt hooks, he was able to free the innominate from its surroundings, and to pass a catgut ligature round it just before its bifurcation. For several hours the patient remained unconscious, but afterwards rallied, and it was not till this occurred that the dressing of the wound was completed. There was no return of hæmorrhage, the wounds healed perfectly, and the man was discharged well forty-three days after the operation. Dr. Lewtas truly remarks that this operation was performed under critical circumstances, which allowed no opportunity for much consideration, or for any reference to authorities. Had such opportunity been permitted, it is very probable the operation would never have been performed, for the statistics of ligature of the innominate artery are not promising, Mr. Erichsen tabulating seventeen instances with only one recovery. This case illustrates the manner in which Indian medical officers in remote districts are often called upon to act altogether on their own opinions and resources. A medical officer in India may be hundreds of miles from any other practitioner, and is, therefore, obliged to assume the full responsibility of all cases presented. Dr. Lewtas is to be congratulated on his prompt and successful action.

### THERAPEUTICS.

### Antipyrin.

Dr. Rayner Batten and Mr. T. J. Bokenham, publish a paper entitled "A Contribution to our Knowledge of the Physiological Action of Antipyrin," especially with regard to the action of the drug in migraine and kindred diseases. Experiments were made on various animals, and from these,

* *Lancet*, June 22, 1889.

and their observations the experimentalists arrive at the somewhat lame conclusion that the main, if not the sole, action of antipyrin is due directly or indirectly to its influence on the nervous system; mainly on the spinal cord, but also on the brain and motor nerves. The tract of the spinal cord specially affected by antipyrin was not, however, discovered. Now the principal effect of antipyrin is to lower the temperature of the body, and hence it has been much used in febrile complaints. How it could lower the temperature excepting through action on the nervous system is not, in the present state of our knowledge, clear; but how it acts on the nerves we do not know, and the experiments mentioned above have not increased our knowledge on this subject to any appreciable extent. Antipyrin, kairin, and other therapeutical agents of the same class not only lower the bodily temperature, but they often excite nausea and sickness, for the explanation of which, as a cause, we are obliged to fall back on that unsatisfactory term "nervous influence." The authors of the paper mentioned remark: "The importance of being able to localise the action of a drug to a special tract of tissue cannot be over-estimated." This may be fully admitted, and if further work should prove it to be possible it will, as the authors observe, "go far to render the use of drugs in diseases of the nervous system a more rational proceeding."

While on the subject of antipyrin, it may be mentioned that Dr. R. Quaita, Milan, recommends it in whooping cough as the "sovereign remedy." He gives twenty-five centigrammes of antipyrin in twenty-five grammes of syrup of orange peel, with an equal quantity of distilled water, in four doses, in twenty-four hours, increasing the dose according to indications.

But, as with other drugs, so with antipyrin, there are persons who cannot tolerate it. A case of this kind is recorded in the *British Medical Journal* of June 15, 1885, when a moderate dose immediately excited burning in the throat, dizziness, loss of vision, and dyspnœa.

At a recent meeting of the Société de Thérapeutic, Dubousquet also advocated the treatment of whooping cough by antipyrin, which he states shortens the course of the disease. He prescribes five to fifteen grains per day to children under three years of age. To a child of five, a five-grain dose may be given after each fit of coughing, till three or four doses have been taken. The medicine may be administered in raspberry syrup or Vichy water.

### Antifebrin.

A case is reported by Dr. Wilding, of Bristol, where ten grains of antifebrin produced toxic effects. The patient was a young man suffering from acute pulmonary tuberculosis. In fifteen minutes after taking the antifibrin he was "all in a glow." In half an hour he became excessively red all over the body, and perspired profusely. In another half hour the temperature fell from 103·1deg. to 98·3deg. The heart sounds were feeble, and the pulse could not be felt at the wrist. Respiration was of a sighing character. The pupils were dilated. Four hours after taking the antifibrin he was apparently moribund. Recovery, however, took place under ammonia by the mouth, hot bottles to the axillæ and feet, and mustard poultices on the chest. The patient had no recollection of what had happened. Two days later two grains of antifebrin were tried, with similar, although much slighter results. It would seem that all agents which are capable of reducing the temperature of the body, occasionally excite unpleasant toxic symptoms—not even excepting quinine. Such effects can only be attributed to some existing constitutional idiosyncrasy. We have similar effects produced in some persons, not only by medicines, but by various kinds of food. There are people who cannot take any kind of shell fish, although it may be quite fresh and good, without suffering from nettle rash. Celery has also been known to excite a similar eruption. As regards the administration of medicines, most practitioners before prescribing mercury, or ipecacuanha, or opium, would enquire if any constitutional idiosyncrasy existed against these drugs, and it now appears necessary to exercise similar caution when prescribing medicines which lower temperature. And as persons are often not aware of the existence of any peculiar idiosyncrasy, it would be well if the additional precaution were adopted of giving a minimum dose at first. It is only by such care that the occasional occurrence of unpleasant results can be avoided.

### RECENT RESEARCH.

### Microbes and Poisons.

The Croonian lectures on "The Relationship Between Chemical Structure and Physiological Action" deserve earlier attention than we have been able to afford. These lectures were delivered by Dr. Lauder Brunton before the Royal College of Physicians, and are marked by exhaustive scientific knowledge and close scientific reasoning, as, indeed, was to be expected in anything from the pen of Dr. Lauder Brunton. It is, however, impossible to do justice to them in a short notice of this kind. They require to be studied in detail, and we should be glad to see them reprinted in an easily accessible form. At the present time we must content ourselves with remarking on one or two points. One of the most interesting part of the lectures is that which treats of the nature of "diseases, microbes, and poisons." Disease was formerly looked upon as something apart from the patient, but we now regard the patient and the disease as living in the same way and influenced by similar conditions. For a long time microbes were looked upon as directly causing disease by affecting the tissues. But since chemical investigation has been employed we are beginning to understand that microbes prove injurious by forming poisons. In 1866, Panum demonstrated that the poison which occurs in putrefying meat is a chemical substance. In 1870, Selmi brought forward the chemical nature of the poisonous products which result from the decomposition of albuminous matter by microbes. In 1873, Dr. Brunton pointed out the striking resemblance between the symptoms of cholera and those of poisoning by muscarin. Since then the researches of Koch and the methods he has introduced have given an enormous impetus to the study of bacteriology, and the isolation by Nencke of a definite ptomaine has put the chemistry of putrefaction on a new basis. There appears now no doubt from the experiments of Dr. Lander Brunton, aided by Dr. Macfadyen, that microbes do form ferments or poisons through which they prove injurious.

### Filaria.

Brigade Surgeon Sibthorpe, Professor of Surgery at the Medical College of Madras, has published an interesting paper "on the adult male *Filaria sanguinis hominis*," * of which drawings are given. Dr. Sibthorpe, however, does not say anything about previous workers in the same field of research. It is, therefore, advisable to mention that the *filaria sanguinis hominis* was first discovered in 1871 by Dr. Lewis, of the Indian Medical Service, in the milky fluid from a lymph scrotum. And in 1872, Lewis also found filaria in the blood. The worm is found in the blood in the shape of an attenuated thread-like creature of a whitish colour, with smooth cuticle or sheath, devoid of transverse marking, but possessing an oral and anal aperture. Sibthorpe supplements this by describing a male and a female worm. An account of the life cycle of this *filaria* has been given by Manson, who regards the mosquito as the intermediary. The mosquito, feeding at night, imbibes filaria with the

* *British Medical Journal*, June 15, 1889.

blood of its human victim, and then retiring, digests its meal, but fails to digest the filaria it has swallowed. The mosquito next deposits its eggs in water, and the filaria also escapes, having, while in the body of the mosquito, developed a boring apparatus. Filaria thus surviving, are swallowed with water by human beings. From the human stomach the filariæ bore their way into the thoracic duct or some lymphatic, where they breed, and the progeny pass into the circulation, to await the chance of a friendly mosquito. Whether all this is fact or theory, it is certain that the *filaria sanguinis hominis* is closely connected with several maladies, the principal of which are certain forms of nematuria, chyluria, elephantiasis, and lymph scrotum, which is really another form of elephantiasis. The latter maladies are characterised by hypertrophy of tissue, and it has been supposed that the filaria, or their embryos, act as emboli in the lymphatics, whence ensues stasis of lymph, regurgitation of lymph, and partial compensation by anastamoses of lymphatic vessels. This brings about hypertrophy of tissue, and may go on to elephantiasis. Dr. Sibthorpe does not tell us all this, but he mentions that the filaria is not always found in cases of elephantiasis. Manson, however, explains this by saying that the habits of the filaria are nocturnal, to suit those of the mosquito, and he regards it as an instance of the wonderful adaptive power of Nature. During the day, Manson says, the filaria rest in the minute branches of the pulmonary artery, and thus periodicity of presence is accounted for.

We have not referred above to Manson's limiting the noxiousness of filaria to occasions when immature birth of the ova of the filaria takes place, as we did not desire to further complicate the question by considering this absurdity, for it may be safely presumed that immature birth or miscarriage is no more usual among nematoid worms than it is among human beings, and that it is therefore an abnormal accident. The theory which attributes irritation and inflammation to filaria or their ova, acting as foreign bodies, is one which may be reasonably considered. But the theory which requires immature expulsion of the ova because the ova are larger in diameter than the filaria, and so are more likely to create irritation, is one which we do not think it necessary to consider.

In all this mass of fact and theory several clearly-established items stand forth. First, there is such a worm as the *filaria sanguinis hominis*. Secondly, it has been found in human blood, and in the diseased tissues and secretions of certain maladies. Thirdly, such maladies have existed without the presence of the worm, or at least where it could not be discovered. Fourthly, the worm has been found in the body of mosquitos. Whether the worm is the cause of the different maladies with which it is found in connection, has yet to be proved. Filariæ, especially in China, are found in the blood of many persons not suffering from any apparent disease. As the matter at present stands, we cannot agree with Manson, who stated, "If people in countries where filaria is endemic would cover their water jars with netting sufficiently fine to keep out mosquitos, they would never get filaria in the blood, or the disease it produces, elephantiasis."

### NEW DRUGS, APPLIANCES AND THINGS MEDICAL.

### SKIN DISINFECTION IN SCARLET FEVER, BORO-GLYCERIDE.

It seems to be now a tolerably well-established fact that the infectious stage of scarlet fever begins with the shedding of the epidermis, or "peeling," after the acute symptoms have abated. The process may consist in the falling of a number of tiny scales, or the skin may come off in flakes of various sizes, in some cases making complete casts of the hands or feet. The recognition of the fact that "desquamation" is the chief agent in spreading the disease is one of the greatest modern advances in the preventive treatment of this malady. For on it is founded a rational plan of dealing with the germ-laden skin. By the simple process of inunction with an antiseptic oil or ointment the scales are mechanically prevented from floating off into the surrounding atmosphere. It is a common plan with many medical men to use inunction over the whole body for three days after desquamation commences: a warm antiseptic bath is then given and the patient pronounced free from contagion. But there are certain objections to the use of carbolic acid for this purpose. The smell is disagreeable, and the drug is often irritating to a tender skin, which, be it remembered, has just recovered from a more or less severe inflammation. Moreover, these volatile substances quickly evaporate from the surface of the body, so that their action is limited in duration. A compound which answers the purpose well, and is highly spoken of by medical officers of fever hospitals, is the Barff boro-glyceride. This simple antiseptic is pleasant and grateful to patients, and makes a nearly perfect application for the skin of scarlet fever. But whatever application the practitioner may prefer, there can be no doubt that the combined principles of mechanically binding down, and of disinfecting the shedding scales, will be the main lines of future preventive measures. The disease is treacherous, and hard to combat. Infection will lurk for years in clothes, in books, in rooms, and in all sorts of unlooked-for places. The theory of the germs being locked up, so to speak, in the shed epithetial scales accounts for many otherwise obscure cases of infection. But although that be the chief, it is by no means likely it is the only method in which the disease may be communicated. The breath and the various excreta of the body should also be regarded as probable channels for the elimination of the poison, and strict precautions should be taken in accordance with that view. The stamping out of scarlet fever presents one of the great future problems of preventive medicine. There is hardly any disease that has a more important bearing on the welfare of the community, both from its direct bills of mortality and from its remoter effects. Many cases of kidney disease owe their origin, in the first place, to scarlatina. To a lesser degree, the same statement is true of numerous affections of the heart—chiefly valvular—and also of the lungs. Scarlet fever often spreads with fearful rapidity in our crowded centres of population, and an outbreak at present raging in Birmingham has filled all the infectious hospitals in the town to overflowing. Fortunately, the inhabitants are working well with the authorities, and parents seem anxious to get their children off to the hospital as quickly as possible. Medical men also send in early notification of each case, for which service they receive a fee. However, before any great headway can be made against the spread of infectious fevers, it will be necessary to make notification compulsory, and to give local boards full powers to isolate, disinfect, and otherwise deal with infected patients. Quite lately a Birmingham publican was fined forty shillings and costs for allowing his son to run about the street while peeling after scarlet fever. The facts of the case seem to have been brought under the notice of the inspector of nuisances by the merest chance. Indeed, while patients are allowed to remain in their own homes, there is very little chance of anything like efficient supervision. Legislation may do much in helping to marshal the scientific forces which will one day assuredly banish scarlet fever and other allied diseases from off the face of the earth.

